# Endoscopic Retrograde Cholangiopancreatography in Children: Nine Years' Experience at Santa Fe Foundation in Bogota, Colombia

**DOI:** 10.7759/cureus.41835

**Published:** 2023-07-13

**Authors:** Sofía R Gómez, Alvaro A Trujillo Ceballos, Gonzalo A Montaño Rozo, Jaime Solano Mariño, Ailim M Carias Domínguez, José F Vera Chamorro

**Affiliations:** 1 Pediatrics, Fundación Santa Fe de Bogotá, Bogotá, COL; 2 School of Medicine, Universidad de los Andes, Bogotá, COL; 3 Pediatrics and Child Health, Fundacion Santa Fe de Bogotá, Bogotá, COL; 4 Gastrointestinal Surgery, Fundación Santa Fe de Bogotá, Bogotá, COL; 5 Pediatric Gastroenterology, Fundación Santa Fe de Bogotá, Bogotá, COL; 6 Pediatric Gastroenterology, Fundación Santa Fé de Bogotá, Bogotá, COL

**Keywords:** endoscopic retrograde cholangiopancreatography, pancreaticobiliary disease, biliary disease, pancreatic disease, children, pediatrics, endoscopic retrograde cholangiography

## Abstract

Introduction: Experience in the use of diagnostic and/or therapy of endoscopic retrograde cholangiopancreatography (ERCP) in children is limited. This is due to the underdiagnosis of pancreaticobiliary disease in the pediatric population and specialist personnel in this procedure.

Objective: To determine the safety and success rate of ERCP in children at Hospital Fundación Santa Fe de Bogotá between January 2007 and June 2015.

Methodology: This was an observational, descriptive, retrospective case series study of patients under 18 years, who underwent ERCP between January 2007 and June 2015. The following variables were analyzed: indication, duration, type of procedure, rate of success, and complications.

Results: A total of 30 patients were included, in whom 65 ERCP procedures were performed. Successful cannulation was achieved in 52 of the 65 procedures (80%). Among the complications that occurred, there were four cases of pancreatitis (6.2%), two cases of bleeding (3.1%), and one case of bacteremia (1.5%), and in most cases (58 in total, 89.2%), there were no complications.

Discussion: The pediatric gastroenterology group of the Fundación Santa Fe de Bogotá has obtained good results in performing ERCP in the pediatric population with a success rate of 80% associated with a null mortality rate. There is enough literature available to conclude that performing ERCP in the pediatric population maintains an adequate success rate and a low complication rate. In all the studies evaluated, a null mortality rate was found, so it is considered that this procedure is safe in patients under 18 years of age.

## Introduction

Pancreaticobiliary diseases have a low prevalence in the pediatric population, and for this reason, they have been less studied than other populations, which means information regarding their diagnosis and management is quite limited. Endoscopic retrograde cholangiopancreatography (ERCP) is the procedure indicated for the diagnosis and treatment of biliary tree and pancreatic pathologies in adults and children [1,2]. Since there is limited information about this procedure in children, there are no requirements for its performance and maintenance established by the guidelines of the American Society for Gastrointestinal Endoscopy, so the same indications are used as in adults [3-6]. This may be related to the limited experience in pediatric patients and the fact that the data found in the literature describe a small number of patients, which has been associated with the lack of a defined consensus on its indications, safety profile, success rate, and complication rate [7]. As a consequence, the research objective is based on evaluating indications, type of procedure, findings, success rate, and complications of ERCP at the Fundación Santa Fe de Bogotá.

Although the prevalence of pancreatic-biliary diseases in children is lower than in adults (the prevalence of biliary lithiasis in adults is 6-9% while in children it is 0.15-1.9%) [8,9], it has a high impact on the quality of life of the child who suffers from it since they usually need more than one intervention to achieve control of their pathology and this limits their expected development.

The endoscopist who will perform the procedure, according to the guidelines of the American Society for Gastrointestinal Endoscopy, must have performed 100 endoscopies and at least one ERCP weekly to have adequate skill in the procedure [3]. However, recently, the number of procedures has increased to 200 and it is required that in 80% of the cases, he/she has been able to perform the cannulation of the common bile duct [4], to increase the safety and quality of the technique. However, few pediatric patients need ERCP and therefore there is not enough experience to meet the requirements of being a pediatric endoscopist with ERCP experience [10-15]. The anesthesiologist, who must be present at the procedure, must choose between sedation and general anesthesia depending on the individual benefit and risk of each patient; mainly sedation with meperidine or fentanyl and diazepam or midazolam is administered [9]. Antibiotic prophylaxis is recommended due to the risk of bacteremia, and it is effective in preventing cholangitis and septicemia in complicated patients (defined as incomplete or unsuccessful biliary drainage) [7]. Prophylaxis is recommended in the presence of cholangitis, biliary obstruction, and/or a history of liver transplantation [14].

It has been seen that patients between one year of age and adolescents have successful cannulation in 95% of the procedures; in neonates, the data are very different, varying between 27% and 95%, being the duodenal malrotation and the inability of the professional to achieve cannulation the limitations for the procedure to be effective [8]. The most important reported complications are pancreatitis, infections, bleeding, and perforation [6,7].

The most described indications in children under 18 years of age are biliary disease and pancreatitis. The indications of biliary disease are divided into diagnostic and therapeutic. The diagnostic indications are mainly neonatal cholestasis and investigation of abnormal findings in other examinations. Therapeutic indications include biliary obstruction and postoperative complications of laparoscopic cholecystectomy. Indications in pancreatitis are for acute and recurrent pancreatitis where an anatomical cause is suspected [16,17].

## Materials and methods

A retrospective, descriptive, observational study was conducted. The study population was pediatric patients (≤18 years) who had undergone ERCP between January 2007 and June 2015 at the Hospital Universitario Fundación Santa Fe de Bogotá (HU-FSFB). We performed a non-probabilistic sampling of patients who met the only inclusion criterion, which was defined as patients who had undergone ERCP between January 2007 and June 2015 at the Hospital Universitario Fundación Santa Fe de Bogotá. No exclusion criteria were defined.

The information from the medical records of the patients meeting the inclusion criteria was collected from the electronic records of the HU-FSFB HIS-ISIS® information system. The information collected was grouped into 15 categorized variables, including patient number, number of procedures performed on the patient, identification number, age, sex, the total number of procedures performed, indication 1 (biliary or pancreatic), indication 2 (specific diagnosis), history, type of anesthesia used, type of antibiotic used, findings in the procedures, type of therapeutic intervention performed, successful cannulation, and complications secondary to the procedures. Each of these variables is defined in Table 1.

**Table 1:** Variable definitions

Variable number	Name	Conceptual definition of the variable	Operational definition (indicator)	Scale of measurement
1	Patient number	Number used for numerical sequencing of patients	Patient number in order of inclusion in the study	Open question/numbers only
2	Procedure number	Number used for numerical sequencing of the procedures included in the study	Procedure number in order of inclusion in the study	Open question/numbers only
3	Patient ID	Unique number used for the identification and recognition of patients in the study	Identification number of the patient's medical record	Open question/numbers only
4	Age	Time a person has lived	Age at the time of intervention in years + (months/12)	Open question/numbers only
5	Sex	Sexual identity of living beings. Consciousness that a person has of being him/herself and different from others	Sexual identity of participants	Male = 1, female = 0
6	Number of attempts	Total number of procedures in a patient	Total number of procedures in a patient	Open question/numbers only
7	Indication 1	Procedure indication	Indication	Pancreatic = 0, biliary = 1
8	Indication 2	Other indications for the procedure different than “indication 1”	Other indication	Open questions/letters only
9	Medical history	Relevant previous medical history	Medical history	Open questions/letters only
10	Anesthesia	Type of anesthesia during the procedure	Anesthesia	General = 0, sedation = 1
11	Antibiotic	Type of antibiotic prior to the procedure	Antibiotic	Ampicillin/sulbactam = 1, gentamicin = 2, clindamycin = 3, piperacillin/tazobactam = 4, none = 0
12	Findings	Intraoperative findings	Findings	Open questions/letters only
13	Therapeutic procedure	Type of therapeutic procedure performed during the procedure	Procedure	Papillotomy = 1, stent insertion = 2, stent removal = 3, biloma drainage = 4, gallstones removal = 5, none = 0
14	Successful cannulation	Cannulation procedure carried out satisfactorily	Cannulation	Yes = 1, no = 0
15	Complication	Secondary complications to the procedure	Complications	Pancreatitis = 1, bleeding = 2, bacteremia = 3, perforation = 4, none = 0

The information was collected through a questionnaire entered in a database in Excel 2010 (Microsoft Corporation, Redmond, WA), and a telephone follow-up was carried out with the patients' guardians in case additional information was required that could not be collected by reviewing the clinical history. Only the researchers had access to the database, which was checked every 15 days by the study investigators to eliminate possible biases and maintain the veracity of the data. Any doubts regarding the filling out of the variables in the database were consulted with the patient's treating physician to ensure that they were filled out optimally. To reduce the loss of data, each time an ERCP was to be performed, a questionnaire was filled out with the variables previously described before the patient left the institution.

For the statistical analysis, measures of central tendency and dispersion were used to determine quantitative variables, and frequency measures were used for qualitative variables. Statistical analysis was performed with STATA Special Edition 11.1 (StataCorp LLC, College Station, TX).

## Results

A total of 30 patients were obtained, in whom a total of 65 ERCP procedures were performed. Of these 30 patients, 11 were male (36.7%) and 19 were female (63.3%). The ages of the patients ranged from 1.5 to 15.8 years, with an average age of 8.18 years. The number of procedures required by each patient varied, with one procedure being the minimum and 10 procedures being the maximum. Of the 65 procedures performed, 48 (73.8%) involved schoolchildren, 11 (16.9%) adolescents, four (6.2%) preschoolers, and two (3.1%) infants.

Regarding the indications, 36 procedures (55.4%) were performed for biliary indications, and 29 procedures (44.6%) for pancreatic indications. As the diagnosis for which the patients were taken for procedures, the most frequent indication was chronic recurrent pancreatitis in 23 (35.4%) procedures, followed by 15 (23.1%) biliary strictures, six (9.2%) suspected common bile duct cysts, four (6.2%) suspected post hepatectomy biliary leak, four (6.2%) suspected choledocholithiasis, and four (6.2%) cholestasis; other indications found to a lesser extent were narrowing of the pancreatic duct, pancreas divisum with recurrent pancreatitis, and icteric syndrome. In a total of 52 of the 65 procedures (80%), successful cannulation was achieved. Among the complications that occurred, there were four cases of pancreatitis (6.2%), two cases of bleeding (3.1%), and one case of bacteremia (1.5%), and in most cases (58 in total, 89.2%), there were no complications.

During each ERCP, various therapeutic procedures were performed, with stent insertion being the most frequent, performed in 33 (27%) of the procedures, followed by papillotomy, which was performed 29 times (23.8%), and 24 (19.7%) stent removals, 14 (11.5%) stenosis dilatations, nine (7.4%) stone extractions, and six (4.9%) occasions where no therapeutic procedure was performed. Sclerotherapy and T-tube removal were performed two times each (1.6%), and biloma drainage, stent accommodation, and a failed procedure occurred one time each (0.8%).

Regarding the type of anesthesia and prophylactic antibiotic, according to institutional protocols, all children were managed with general anesthesia and ampicillin sulbactam according to their weight; therefore, no significant variables were considered.

## Discussion

Although case reports have demonstrated the feasibility of ERCP in youth and children, there is not much information on the safety and outcomes of these interventions in a significant number of patients. The largest study was done in 1993 by Brown et al. [1], which included less than 100 patients. The next large study was a systematic review published in 2018 by Keane et al. [16], in which patients were studied from 1992 to 2014 where 87 ERCPs were performed in 66 patients.

Based on our study with the information decoded, we know that a total of 30 patients were treated at the Fundación Santa Fe de Bogotá between January 2007 and June 2015, of which 63.3% (19) correspond to female patients and 36.7% (11) to male patients, which corresponds to the gender behavior described in different studies in which the prevalence of the female gender is reported between 57% and 73% of the patients treated [15,18,19].

The age range of the intervened patients corresponds to 1.5 and 15.8 years with a mean age of 8.18 years, which is in agreement with what is described in the literature, where mean ages of 9.89, 13.5, 14.6, and 15 years are reported [15,20-22].

Regarding the age group, it was found that the most operated patients were schoolchildren with a total of 48 procedures, which corresponds to 73.8% of the ERCPs performed, while the least operated patients were infants with only two procedures, i.e., 3.1%. This is in agreement with what has been found in the literature, which reports that the majority of interventions are performed in school patients, i.e., between six and 12 years of age [15,19-21].

Regarding the indications described above in the results section, we found that they are not consistent with other studies, such as the one conducted by Quintanilla et al. in which the main indication for performing ERCP was abdominal pain with elevated liver enzymes, followed by post liver transplant complications and evaluation of biliary tract stenting. Similar behavior was observed in Cerezo-Ruiz et al.'s study, in which 33.3% of the indications corresponded to post-transplant liver complications, 27.77% to suspected biliary obstruction, and 22.2% to the evaluation of pancreatitis. Among the indications found in different studies, we also found biliary obstruction, pancreatitis, pancreatic duct disruption, and biliary leakage [15,19,21].

To analyze the success rate, the variable "successful cannulation" was defined as "successful procedure" and we found a success rate of 80%, since of the total number of procedures performed, 80% (52) achieved successful cannulation, while 20% (13) did not. In different studies, the success rate was defined as the achievement of cannulation and repletion of the desired conduit, finding success rates ranging from 76.47% to 100%, which agrees with that found in the present study [15,21].

A total of seven patients presented complications, which included bacteremia (one case), bleeding (two cases), and pancreatitis (four cases), thus concluding that the complication rate was 10.8% with pancreatitis (6.2%) being the most common complication. The above is in agreement with the literature, which reports that complication rates range between 4% and 8.3%, with mild acute pancreatitis as the main complication with a rate ranging between 2% and 5.7% [15,19,21].

## Conclusions

It can be concluded that the pediatric gastroenterology group of the Fundación Santa Fe de Bogotá has obtained good results in the performance of ERCP in the pediatric population based on a success rate of 80% associated with a null mortality rate. However, it presents a complication rate of 10.8%, which exceeds the range of 4% to 8.3% reported in the literature. This is probably due to the indications associated with the performance of the procedure. In most studies, the main indication was complications associated with liver transplantation, while in our case series, no procedure was performed under this indication.

There is enough literature available to conclude that the performance of ERCP in the pediatric population maintains an adequate success rate and a low rate of complications. In all the studies evaluated, a null mortality rate was found, so this procedure is considered safe in patients under 18 years of age. Subsequent studies could be performed with a larger population, and multicenter studies are needed to obtain an analytical observational study in which associations with complications and success rate could be investigated to better define the safety profile of ERCP in children.
